# Editorial: Multiscale cancer modeling, *in silico* oncology and digital (virtual) twins in the cancer domain

**DOI:** 10.3389/fphys.2025.1614235

**Published:** 2025-05-26

**Authors:** Georgios S. Stamatakos, Maria Angeles Perez, Ravi Radhakrishnan

**Affiliations:** ^1^ In Silico Oncology and In Silico Medicine Group, Institute of Communication and Computer Systems, School of Electrical and Computer Engineering, National Technical University of Athens, Athens, Greece; ^2^ Multiscale in Mechanical and Biological Engineering, Aragon Institute of Engineering Research (I3A), Aragon Institute of Healthcare Research (IIS Aragon), University of Zaragoza, Zaragoza, Spain; ^3^ Department of Bioengineering, University of Pennsylvania, Philadelphia, PA, United States

**Keywords:** *in silico* oncology, *in silico* medicine, mechanistic multiscale modeling, digital twin, virtual twin, cancer, artificial intelligence, machine learning

*In silico* oncology, a pivotal branch of the fast evolving *in silico* medicine, is a scientific, technological, and progressively clinical discipline that aims to support cancer prevention, diagnosis, prognosis, monitoring and patient-specific optimization of clinical interventions or simulating clinical studies by conducting *in silico* experiments, i.e., experiments on a computer. *In silico* experiments utilize models or technologically integrated digital (virtual) twins of parts of - or the entirety of–the human body, and eventually its environment, as well as these models’ biological behavior and/or interactions with eventual intervention(s). The models utilized can be based on mechanistic multiscale modeling and simulation and/or artificial intelligence (AI) modeling including machine learning. Each model must undergo a strict clinical validation and certification before being exploited in a real clinical setting.

**FIGURE 1:**
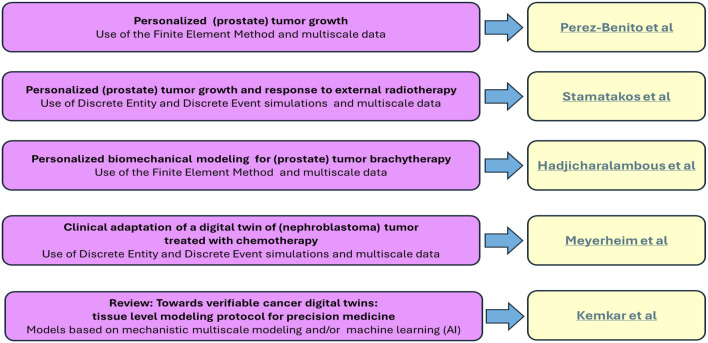
Cancer digital (Virtual) twins. A brief summary of the key contents of the five papers included in the collection.

The following five papers in this Research Topic provide diverse novel approaches, new findings, new insights and new protocols for the development of verifiable cancer digital twins (See Figure 1).

Perez-Benito et al. present a novel frame for the creation of a personalized prostate cancer digital twin, by integrating clinical MRI data, including the prostate and tumor geometry and the initial cell distribution and vasculature. Their approach simulates and predicts temporal tumor growth in the prostate by using the Finite Element Method. Tumor growth dynamics is coupled with oxygen transport, whereas cellular processes, including proliferation, differentiation, and apoptosis are also incorporated. Additionally, their approach simulates the dynamics of the prostate-specific antigen (PSA), thus allowing the evaluation of tumor growth through the patient’s levels of PSA. To calculate the model parameters, a process based on multi-objective optimization is applied and the optimal parameters for two patients are simultaneously adjusted. The proposed frame is validated using data from four patients with several follow-up MRIs. The current version of the model predicts the growth of both prostate and tumor volume as well as serum PSA levels.

Stamatakos et al. present a novel multiscale mechanistic simulation model as the core of a personalized digital twin of prostate tumor growth and its response to external radiotherapy schemes. Their mathematical approach is based on the use of discrete entities and discrete events. Following technical verification, an adaptation approach to clinical data is outlined. The German clinical study HypoFocal-SBRT has provided the multiscale data exploited. A sensitivity analysis has been implemented. The impact of model parameters including cell cycle duration, apoptosis rate of living and progenitor cells, fraction of dormant stem and progenitor cells that reenter the cell cycle, fraction of stem cells performing symmetric division, fraction of cells entering the dormant phase following mitosis, alpha and beta parameters of the linear-quadratic radiobiological model and oxygen enhancement ratio has been studied. The model has been shown to agree at least qualitatively with available experimental and clinical knowledge. The latter has dictated the next steps towards a thorough clinical validation of the model, its technological integration and its eventual certification and clinical translation. A brief historical review of the formal emergence of *in silico* medicine (in 2002) and digital twins in oncology and beyond (in 2007) is also provided.

Hadjicharalambous et al. introduce a data-driven *in silico* modeling approach to simulate the biomechanics of prostate brachytherapy through the use of the Finite Element technique. Comprehensive sets of magnetic resonance and transrectal ultrasound images collected before, during and after brachytherapy are utilized for the model personalisation. The therapeutic intervention is simulated through the sequential insertion of multiple catheters into the prostate gland. The medical imaging data is also exploited for model evaluation, therefore showcasing the potential of the proposed *in silico* procedure to be used pre- and intra-operatively in the clinical environment.

Meyerheim et al. delineate an *in silico* study aiming at clinically adapting a personalized pediatric nephroblastoma digital twin i.e., the Nephroblastoma Oncosimulator. The following data categories from three patients enrolled in the SIOP 2001/GPOH clinical trial have been exploited: T2-weighted MRI scans, chemotherapy treatment plans, and post-surgical histological profiles. Each selected patient represents a distinct clinically assessed risk group. The clinical adaptation of the digital twin to these datasets is investigated in this paper. The aim of the latter is to obtain appropriate value distributions of the model input parameters that allow a nearly accurate prediction of tumor volume reduction due to preoperative chemotherapy. Distributions of the total cell kill rate have been derived for one patient from each risk group, i.e., for the low, the intermediate and the high risk groups. Statistically significant differences have been observed between the high-risk group and both the low- and the intermediate-risk groups.

Kemkar et al. have compiled an extensive literature review that discusses recent advancements in *in silico* methodologies for precision oncology, focusing on the potential of cancer digital twins to drastically reinforce patient-specific decision-making in the clinical setting. The authors review several computational approaches to the creation of patient-informed cellular and tissue-level models for cancer. They also propose an *in silico* framework that exploits agent-based modeling as an effective conduit to integrate cancer systems models that encode signaling at the cellular level with digital twin models predicting tissue-level response in a tumor microenvironment customized to the actual patient’s data. Additionally, they discuss machine learning (artificial intelligence or AI) approaches to creating surrogates for these complex mathematical models. These surrogates can potentially be utilized to conduct sensitivity analysis, verification, validation, and uncertainty quantification. The latter are especially important for tumor studies due to their dynamic nature.

In summary, digital or virtual twins are becoming pivotal in oncology. Cancer digital twins facilitate personalized cancer care by simulating disease progression and treatment responses, enhancing decision-making and therapeutic outcomes. The transformative potential of digital twins in healthcare has been emphasized both in the European Union (EDITH, EMA, Avicenna Alliance - Cancer and In Silico Oncology Task Force) and the United States (National Academies, ASME). Collectively, these endeavors highlight a global commitment to integrating digital or virtual twins into medicine and especially oncology. By fostering collaboration between engineering, medical, and regulatory communities, these *in silico* initiatives are expected to strengthen personalized medicine and accelerate the development of effective cancer therapies.

